# The Effect of Neuro-Physiotherapy on Gross Motor Function in a Male Child With Spastic Diplegic Cerebral Palsy: A Case Report

**DOI:** 10.7759/cureus.29310

**Published:** 2022-09-19

**Authors:** Pallavi Harjpal, Anushka Raipure, Rakesh K Kovela, Moh'd Irshad Qureshi

**Affiliations:** 1 Physiotherapy, Ravi Nair Physiotherapy College, Datta Meghe Institute of Medical Sciences, Wardha, IND; 2 Physiotherapy, NITTE Institute of Physiotherapy, NITTE (Deemed to be University), Mangalore, IND; 3 Neuro-Physiotherapy, Ravi Nair Physiotherapy College, Datta Meghe Institute of Medical Sciences, Wardha, IND

**Keywords:** case report, physiotherapy, spastic diplegia, rehabilitation, cerebral palsy

## Abstract

Cerebral palsy (CP) is a condition caused by a non-progressive lesion in the developing brain. CP has a wide range of prevalence, ranging from 1.5 to three cases per 1,000 persons. Newborns weighing less than 2,500 grams now account for half of all incidences of CP. Clinical management in physical therapy is a paradigm for enhancing organizational capacity, integrating evidence-based best practices, and enhancing outcomes. This is a case report of a 21-month-old male child with a history of sudden onset of seizure, fever, drowsiness, frothing from the mouth, and up rolling of eyes. He had a global developmental delay with microcephaly and breakthrough seizures with anemia under evaluation. Magnetic resonance imaging (MRI) brain revealed the possibility of hypoxic-ischemic insult. The child was managed conservatively using medications i.e., ibuprofen (7.5mL/6hrly), cephalosporin (450mg/day in divided doses), and phenytoin. Physiotherapy management was provided with integrative approaches including Neurodevelopmental treatment principles, passive stretching, static weight-bearing exercises, and task-oriented approaches. The evaluation was done using the Modified Ashworth Scale and Gross Motor Function Measure-88 (GMFM-88). Early physiotherapy with integrative approaches helps in the improvement of gross motor developmental milestones in children with Spastic diplegic CP.

## Introduction

Cerebral palsy (CP) is a syndrome caused by a non-progressive lesion in the developing brain. CP has a wide range of prevalence, ranging from 1.5 to three cases per 1000 persons [1]. Neonates with low birth weight are becoming more common among CP children. Newborns weighing less than 2,500 grams now account for half of all incidences of CP. The incidence of CP follows a distinct social class gradient [2]. While hypoxia of prematurity has long been thought to be a primary etiologic component, current research suggests that there may be other causes, such as circulating toxins from maternal illness, primarily in the urinary tract but perhaps from other sources [3]. Periventricular leukomalacia (PVL), which is characterized by bilateral necrosis of the frontal and parietal periventricular white matter, is the most common brain lesion associated with spastic diplegia [4]. Even though the underlying brain injury is stable, people with spastic diplegia frequently develop issues with tone, posture, and gait. Due to calf muscle spasticity, standing and walking are taught late, with the ankles in an equino-varus position. As a kid grows older, the crouch gait can be caused by gradual stiffness of the hip flexors and hamstrings, making prolonged walking difficult and giving the appearance of neurological degeneration [4]. There are several causes of the illness, including cerebral anoxia, bleeding, infection, and hereditary disorders.

There are various treatment approaches available for children with CP. Traditional exercises used in physical therapy include the passive hip and lower extremity joint range of motion (ROM) and practicing gross motor skills at an age-appropriate level is important. The therapist should additionally promote greater trunk and pelvic mobility as well as active trunk extension which may result in better balance and posture [5]. Aqua-therapy, which involves alternating movements of the lower extremities, and therapeutic riding on horses are additional beneficial treatments [6].

Clinical management in physical therapy is a paradigm for enhancing organizational capacity, integrating evidence-based best practices, and enhancing outcomes. Low birth weight, intrauterine diseases, and multiple pregnancies are the most common risk factors for CP. CP is four to six times more common in children whose birth weight falls below the 10th percentile. Intrauterine infections raise the chance of CP by five times in full-term newborns and by two times in preterm infants [2]. For these patients, there are a variety of therapy options. None of these methods are mutually exclusive, and the bulk of them are mutually beneficial. There is no “magic bullet” for treating children with spastic diplegia; rather, a combination of modalities must be used to achieve the best possible outcome [3].

## Case presentation

Patient information

A 21-month-old male child born from normal vaginal delivery was brought to the outpatient department with a history of sudden onset of seizure four months back, fever, drowsiness, frothing from the mouth, and up rolling of eyes. He had a global developmental delay and breakthrough seizures with anemia under evaluation.

He was a full-term baby without any post-natal complications. On the 10th day of life, the patient was admitted to the local hospital with complaints of fever and difficulty feeding properly, but no abnormality was detected, allowing the discharge with regular follow-up. Immunization history: the patient was immunized till 1.5 years of age, and last received pulse polio.

As narrated by the mother, the child was apparently all right four months back, on a fine morning, he had a convulsion episode at eight in the morning, further developing weakness in his left hand followed by up rolling of eyes and frothing along with the passing of urine. The entire episode lasted for one hour followed by postictal drowsiness and unconsciousness. The child gives a history of falls from the height of approximately four feet two days back. There was no associated loss of consciousness or abnormal behavior. He neither gives a history of discharge/bleeds through ear/nose nor a history of fever, cough/cold, vomiting/loose stools. After one and a half months, the child was again admitted to a nearby hospital with complaints of fever and drowsiness. He was managed with medications, i.e., ibuprofen (7.5mL/6hrly), cephalosporin (450mg/day in divided doses), and phenytoin. He was discharged against medical advice after four days of hospitalization. On discharge, he was active and afebrile with stable vitals. The mother noticed the child was unable to sit independently and was facing difficulty with movement transitions. Standing independently was also not achieved by the child at one year of age hence got he was evaluated last month. The child was bought to the hospital, for further management.

Clinical findings

The baby was ectomorphic built, conscious, cooperative, and well oriented to time, place, and person. On observation, the attitude of the right ankle was in plantarflexion along with mild torticollis to right. A tongue tie was present. On examination, there was increased tone in hamstrings, Modified Ashworth scale- Grade 1+. Muscle power in the upper limbs was high (grade 4) as compared to lower limbs (grade 3) as graded as pediatric muscle testing [7]. Deep tendon reflexes were exaggerated on the lower limb and a plantar extensor was observed on admission. There was tightness of the hamstring, tendo-Achilles, and adductors on both the lower limbs. The child can crawl and come to sitting from supine with good static balance and dynamic sitting balance is impaired along with difficulty standing and walking independently.

Clinical diagnosis

MRI brain revealed the possibility of hypoxic-ischemic insult. The study also revealed gliotic changes in the periodic region, adjacent bilateral high frontoparietal lobes, bilateral corona radiata, bilateral periventricular region, and bilateral thalami. The clinical features suggest a case of spastic diplegic CP.

Physiotherapy functional assessment

The ROM tests for both the upper and lower limbs were normal, and the qualitative assessment of strength for both the upper and lower extremities was good, but the lower extremities were poor to fair. He had developed tightness of bilateral tendo-Achilles, hamstrings, and short adductors. Silfverskiold test was negative. He faced difficulty in independent standing, transitions, and walking. When made to stand and walk with maximum support, the patient walks on toes and with a scissoring gait. On the first day of testing, the MAS grading for hamstrings was 1+ and the Gross Motor Function Measurement (GMFM-88) was 22%.

Physiotherapeutic interventions

The physiotherapeutic intervention is provided in Table 1. No surgical management was done for the tongue tie, tongue movements were taught to the child and the caregiver. The child performed them regularly under the supervision of a caregiver. Now the child is 2.5 years old and there is no tongue tie at present. Similarly, for torticollis, manual stretching was done for a 10-sec hold and three repetitions, thrice a day.

**Table 1:** The physiotherapeutic intervention in detail

Problem identified	Probable cause	Goal Framed	Physiotherapy Intervention
Spasticity of hamstring, adductors, and tendo-achilles.	Spasticity	To reduce the spasticity of the muscle.	Sustained passive stretching of hamstring, tendo-achilles, and adductors (30 secs hold * 3 rep).
Difficulty standing independently from sitting.	Tight muscles of the lower limb and reduced trunk control	Gain trunk control and independent transition	Sit to stand facilitation on bolster along with single leg sit to stand and multidirectional reach-outs in sitting.
Standing and walking transitions	The inactive base of support	Train standing and activation of base	Standing training, in which the child was asked to stand and conduct activities while receiving maximum support at the pelvis and knees. After two more weeks, we began assisting him in taking a few steps with minimal pelvic support. Task oriented approaches were added to facilitate weight shifting.
Difficulty walking independently	Inadequate trunk control and decreased motor control	Gain walking with minimal assistance	Gait training with minimal pelvic support assistance. Stepping forward and reach outs.
Difficulty in stair climbing	Inadequate trunk control	Gain ground clearance and stair climbing	Step up and step-down facilitation with stepper and bolster along with half-kneeling.

Follow-up and outcomes

After six weeks of integrative neuro-physiotherapy approaches including neurodevelopmental treatment principles, passive stretching, static weight-bearing exercises, and task-oriented approaches, the child gained very good proximal stability. There was a reduction in the spasticity and normal tone was achieved by end of four weeks. There was a tremendous improvement in the GMFM-88 score from 22% to 70%. He initiated transitions willingly, but there were some uncontrolled movements, going for an opisthotonos posture. Therefore, for these problems, transitions from kneeling to quadruped and kneeling to half-kneeling were added to activate the base of support of the baby (as per NDT). In kneeling, oblique activities were focused to train the oblique muscles, as gaze stability (during activities) leads to postural stability. The child was able to walk with the good initial contact and minimum assistance from the caregiver. The prognostic plan for the baby is to reduce unwanted movement and increase purposeful movements while transitioning in the next six weeks.

## Discussion

The clinical parameters of the child were consistent with recent studies on spastic diplegic CP. Physiotherapy management was developed using integrative approaches including Neurodevelopmental treatment principles, passive stretching, static weight-bearing exercises, and task-oriented approaches, and evaluated using the Modified Ashworth scale, Pediatric muscle testing, and GMFM-88 in this situation [7].

The child responded well to the treatment. His session compliance was excellent, which we believe was due to the sort of treatment strategy and techniques used, which included playful activities throughout the sessions. Functional goal-oriented approaches were found to be effective in a study by Das and Ganesh [8], our treatment approach is in line with that study. The success of the treatment regimen also goes to the constancy with which the caregiver delivered home exercise programs and hold numerous meetings to address the mother’s concerns [9,10]. Week after week, we saw steady development. Post one month of rehabilitation, the child was able to maintain sitting balance, initiate reach-outs in sitting, and also stand with support from a sitting position. After six weeks, the caregivers were overjoyed to see the youngster walking with a strong trunk and pelvic control and with minimal help. The GMFM-88 score was 70%, with significant improvements in all five aspects compared to 22% on day 1. This significant improvement shows the positive effects of play therapy along with neurodevelopmental treatment [2]. Similar significant results were obtained in a study based on task-oriented neurodevelopmental therapy [11].

Physiotherapy management was developed using neurodevelopmental treatment principles and evaluated using the GMFM-88 in this situation. The findings of a study suggest that an eight-week neurodevelopmental treatment based on posture and balance training is an efficient method for increasing functional motor level and functional independency in diparetic and hemiparetic CPC by enhancing postural control and balance [12]. In the past 10 years, there has been a noticeable rise in the use of exercise-based therapies to enhance postural control in children with CP [13].

Using neurodevelopmental therapy, we gained commendable results in the child. A previous investigation exploring the use of GMFM 88 in conditions other than CP yielded similar results [3,14]. We will publish a long-term follow-up as well as the role of physiotherapy in the acquisition of fine motor activities shortly.

## Conclusions

This case report concludes that early integrative neuro-physiotherapy with a goal-oriented therapeutic regimen like neurodevelopmental treatment principles, passive stretching, static weight-bearing exercises, and task-oriented approaches helps to improve gross motor developmental milestones in children with spastic diplegia. The child showed improvement clinically as well as with the outcome measures. The child was able to walk with minimal assistance after a month and within two months gained independent walking. He gained good trunk and pelvic control post-rehabilitation.
